# Supplementary material for: Illness perceptions of occupational hand eczema in German patients based on the common-sense model of self-regulation: A qualitative study
Source: PLoS One. 2023 May 12;18(5):e0285791. doi: 10.1371/journal.pone.0285791 (PMC10180686; doi:10.1371/journal.pone.0285791)
Supplement: S2 Appendix — (DOCX) [file pone.0285791.s002.docx]

| English version (translated) | |
| --- | --- |
| Opening question | Please tell me, what it's been like with your skin, in your life so far? |
| Identity | How did you notice that your skin was different/had changed?  What does your skin look like?  Please describe the changes in your skin.  To what extent have you noticed other changes in your body? |
| Cause | What do you think caused the changes of your skin?  What causes your skin to change? |
| Course/Timeline | Thinking back to the last few years, how did your skin develop?  How long did the phases last?  When was it?  How do you imagine your condition will develop in the future? |
| Controllability | What will you do when your skin changes/is inflamed?  Thinking back to your medical treatments, what was done when your skin was changed/inflamed? |
| Consequences | Thinking about your everyday life, what role do your skin changes play when you think about your everyday life? |
| Emotional representation | In what way do your skin changes trigger feelings in you? |
| Coherence | In what way are your skin changes comprehensible to you? |

**S2 Appendix: Interview guide**

| German version (original) | |
| --- | --- |
| Erzählimpuls | Beschreiben Sie doch mal, wie das so war mir Ihrer Haut, in Ihrem bisherigen Leben? |
| Identität | Woran haben Sie gemerkt, dass Ihre Haut anders ist/sich verändert hat?  Wie sieht Ihre Haut an den Händen aus?  Beschreiben Sie doch mal die Veränderungen an Ihrer Haut.  Inwiefern haben Sie weitere Veränderungen an Ihrem Körper wahrgenommen? |
| Ursache | Was sagen Sie, wodurch haben sich die Hautveränderungen entwickelt?  Was sorgt dafür, dass sich Ihre Haut verändert? |
| Verlauf | Wenn Sie an die letzten Jahre zurückdenken, wie entwickelte sich Ihre Haut?  Wie lange dauerten die Phasen an?  Wann war das?  Wie stellen Sie sich vor, wird sich Ihre Erkrankung in der Zukunft entwickeln? |
| Kontrollierbarkeit | Was machen Sie, wenn sich Ihre Haut verändert/entzündet ist?  Wenn Sie an Ihre ärztlichen Behandlungen zurückdenken, was wurde gemacht, wenn Ihre Haut verändert/entzündet war? |
| Konsequenzen | Wenn Sie an Ihren Alltag denken, welche Rolle spielen da die Hautveränderungen? |
| Emotionale Representation | Inwiefern lösen Ihre Hautveränderungen Gefühle bei Ihnen aus? |
| Kohärenz | Inwiefern sind Ihre Hautveränderungen für sie nachvollziehbar? |
