# Supplementary material for: Illness perceptions of occupational hand eczema in German patients based on the common-sense model of self-regulation: A qualitative study
Source: PLoS One. 2023 May 12;18(5):e0285791. doi: 10.1371/journal.pone.0285791 (PMC10180686; doi:10.1371/journal.pone.0285791)
Supplement: S3 Appendix — (DOCX) [file pone.0285791.s003.docx]

**S3 Appendix: Category system**

| English version (translated) | | |  | German version (original) | | |
| --- | --- | --- | --- | --- | --- | --- |
| Main Categories | Sub-Categories | Sub-sub-categories |  | Haupt-kategorien | Sub-Kategorien | Sub-sub-Kategorien |
| Cause | excluded cause |  |  | Ursache | ausgeschlossene Ursache |  |
|  | lifestyle |  |  |  | Lebensstil |  |
|  | external factors/irritants |  |  |  | äußere Faktoren |  |
|  | genetic factors |  |  |  | genetische Faktoren |  |
|  | allergy |  |  |  | Allergie |  |
|  | psychosocial factors |  |  |  | psychosoziale Faktoren |  |
|  | other |  |  |  | sonstiges |  |
| Timeline | steady deterioration |  |  | Verlauf | stetige Verschlechterung |  |
|  | steady improvement |  |  |  | stetige Verbesserung |  |
|  | phasic/cyclical |  |  |  | Phasenhaftigkeit |  |
|  | development of hand eczema |  |  |  | Entwicklung des Handekzems |  |
|  | time before occurrence of hand eczema |  |  |  | Zeit vor Auftreten des Handekzems |  |
|  | total duration of hand eczema | duration of dermatologist procedure |  |  | Gesamtdauer des Handekzems | Verfahrensdauer |
|  | context of changes in hand eczema |  |  |  | Kontext von Veränderungen des Handekzems |  |
|  | context of beginning of hand eczema |  |  |  | Kontext des Beginns des Handekzems |  |
| Identity | legitimation as disease | legitimation of severity |  | Identität | Legitimation als Erkrankung | Legitimation des Schweregrads |
|  | label of hand eczema |  |  |  | Bezeichnung des Handekzems |  |
|  | symptoms |  |  |  | Symptome |  |
| Controllability | ability to assess hand eczema |  |  | Kontrollier-barkeit | Einschätzbarkeit des Handekzems |  |
|  | control beliefs (Bandura) |  |  |  | Kontrollüberzeugung (Bandura) |  |
|  | self-efficacy expectation | lack of perceived self-efficacy |  |  | Selbstwirksamkeitserwartung | fehlende Selbstwirksam-keitserwartung |
|  | successful control |  |  |  | Erfolgreiche Kontrolle |  |
|  | attempted or decreasing control | self-harm as measure for relief |  |  | versuchte oder nachlassende Kontrolle | Selbstverletzung als Linderungs-versuch |
|  | lack of control |  |  |  | fehlende Kontrolle |  |
|  | hypothetical measures |  |  |  | hypothetische Maßnahmen |  |
|  | concrete measures | not clear future possible/ previously implemented measures |  |  | konkrete Maßnahmen | nicht eindeutig zukünftig möglich/bisher umgesetzte Maßnahmen |
|  |  | measures possible in the future |  |  |  | zukünftig mögliche Maßnahmen |
|  |  | measures implemented so far |  |  |  | bisherige Maßnahmen |
| Consequences | no consequences |  |  | Konsequenzen | keine Konsequenzen |  |
|  | financial consequences |  |  |  | finanzielle Konsequenzen |  |
|  | physical or psychological consequence (unclear) |  |  |  | nicht eindeutig physische oder psychische Konsequenz |  |
|  | psychological consequences |  |  |  | psychische Konsequenzen |  |
|  | physical consequences |  |  |  | physische Konsequenzen |  |
|  | professional consequences |  |  |  | berufliche Konsequenzen |  |
|  | private consequences |  |  |  | private Konsequenzen |  |
|  | overall coping with hand eczema |  |  |  | Umgang mit Handekzem |  |
| Coherence | justice |  |  | Kohärenz | Gerechtigkeit |  |
|  | sense of coherence | lack of sense of coherence |  |  | Kohärenzgefühl | fehlendes Kohärenzgefühl |
|  | fatefulness |  |  |  | Schicksalhaftigkeit |  |
|  | non-comprehensibility | no thoughts on comprehensibility of disease |  |  | Nicht-Verstehbarkeit | bisher keine Gedanken gemacht über Verstehbarkeit |
|  |  | missing idea of own vulnerability |  |  |  | fehlende Vorstellung der eigenen Vulnerabilität |
|  |  | nescience is induced externally |  |  |  | Glaube an induzierte Unwissenheit |
|  | comprehensibility |  |  |  | Verstehbarkeit |  |
|  | tends to be comprehensible |  |  |  | tendenzielle Verstehbarkeit |  |
| Emotional Representation | shame - embarrassment |  |  | Emotionale Repräsentation | Scham - peinlich berührt |  |
|  | nervousness |  |  |  | Nervosität |  |
|  | caustic |  |  |  | Ätzend |  |
|  | being irritated |  |  |  | gereizt sein |  |
|  | anger |  |  |  | Wut |  |
|  | context of emotional changes |  |  |  | Kontext emotionaler Veränderungen |  |
|  | joy |  |  |  | Freude |  |
|  | sadness |  |  |  | Traurigkeit |  |
|  | disgust |  |  |  | Ekel |  |
|  | anger |  |  |  | Ärger |  |
|  | frustration |  |  |  | Frustration |  |
|  | fear |  |  |  | Angst |  |
|  | being annoyed |  |  |  | genervt sein |  |
|  | panic |  |  |  | Panik |  |
