# Supplementary material for: Illness perceptions of occupational hand eczema in German patients based on the common-sense model of self-regulation: A qualitative study
Source: PLoS One. 2023 May 12;18(5):e0285791. doi: 10.1371/journal.pone.0285791 (PMC10180686; doi:10.1371/journal.pone.0285791)
Supplement: S4 Appendix — (DOCX) [file pone.0285791.s004.docx]

**S4 Appendix: Original German quotes and English translations as presented in publication**

| **German (original)** | **English (translated)** |
| --- | --- |
| **Causes** | |
| „Aber das Waschen ist mir dann auch nicht gut wegen dem Austrocknen und pH-Wert und allem.“ (B19) | "But then washing isn't good for me either, because of the skin drying out and the pH and everything." (B19) |
| „Ich glaube, ja, ich sage mal: Die Anzahl des Handschuhtragens ist, glaube ich, mehr geworden, als früher. Ja, man zieht einfach mehr an. Das lässt sich nicht vermeiden. Manchmal auch doppelt. Also, das ist eine größere Belastung für die Haut, ohne Frage, weil die Abstände zwischen dem Handschuhtragen auch immer kürzer werden.“ (B13) | "The amount of glove wearing, I think, has become more than it used to be. Yeah, you just put on more. There's no way to avoid it. Sometimes even two pairs on top of each other. So, that's a greater burden for the skin, without question, because the intervals between wearing gloves are also getting shorter and shorter." (B13) |
| „Öl, Fett, Kühlschmierstoff, Dielektrikum, alles zusammen auf der Haut. Und dann noch ein Reiniger irgendwo was ablasen und dann den Reiniger über die Hand abreiben, Lappen, weiter. Oh, das brennt aber, dann geh mal Hände waschen.” (B4) | "Oil, grease, cooling lubricant, dielectric, all combined on the skin. And then another cleaner, blowing something off somewhere and then rubbing the cleaner over your hand, rag over it, and so on. Oh, that burns. Well, go wash your hands." (B4) |
| „Natürlich hat man auch Arbeiten, wo man packt was an was, wie gesagt wenn man in einer Werkstatt tätig ist, Schmutz ist vorhanden.“ (B16) | "Of course, you also have working activities where you touch something, like I said, if you're working in a workshop, there's dirt." (B16) |
| „das Problem war dann vor allen Dingen halt an den Gelenken, dass es da halt eingerissen ist, durch die mechanische Problematik die ich halt auch noch auf Arbeit habe, dass ich halt viel heben oder drehen oder greifen muss.“ (B18) | "The problem was mainly the skin on the joints, that it was torn because of the mechanical problems I still have at work, that I have to do a lot of lifting or twisting or gripping.” (B18) |
| „Man ist ja ausgestattet mit tausenden von Cremes. Und das ist, glaube ich, für die Haut auch nicht das Wahre.“ (B11) | "After all, you're equipped with thousands of creams. And I don't think that's the right thing for the skin either." (B11) |
| „Die Kälte, denke ich mal. Also der Winter, die Kälte, die auf jeden Fall. Und beim, bei der, im Sommer, die die Sonne. Halt auch im Urlaub ist klar, so denn kriegt die Haut auch mehr Sonne ab, dann.“ (B28) | "The cold, I guess. Winter, cold, definitely. And [...] in the summer, the sun. Also on vacation, it's obvious, the skin also gets more sun." (B28) |
| „Ich glaube, dass Schwitzen für mich der Hauptpunkt ist. Durch jetzt die ganzen Maßnahmen, durch Corona natürlich vermehrt. Weil, nicht nur Schwitzen an den Händen, sondern am ganzen Körper.“ (B13) | "I think sweating is the main point for me. Increased through all measures now, through Corona, of course. Because, not only sweating on the hands, but on the whole body." (B13) |
| „Ja dieses Konglomerat an viel. Ne? Handschuhe tragen, Hände desinfizieren, auch dieses ständig-. Ich schwitze sofort unter den Handschuhen. Und Hände desinfizieren, also dieses ständig feuchte. Was dann eben eins auf anderem immer wieder mit dazu kommt.“ (B20) | "Yea, it's this agglomeration of a lot of things, you know? Wearing gloves, disinfection of hands, also constantly- When I'm wearing gloves, I sweat immediately. And disinfections. My hands are constantly wet. Again, and again, more and more on top.” (B20) |
| „Also, ob der LKW jetzt in irgendeiner Baustelle gewesen ist, wo ganz Merkwürdiges sich befindet, wo keiner weiß, was es ist. Es lässt sich auch nicht mehr nachvollziehen. […] Es ist nicht nur einfach das Gefühl von Sand in den Augen, sondern auch mehr.” (B1) | „As if the truck has been in some construction site where there is something quite strange on the ground where nobody knows what it is which is not traceable. [...] It's not just the feeling of sand in the eyes, but something else." (B1) |
| „Oder ich habe ein Pferd, ich reite. Da muss ich auch immer gucken, dass ich Handschuhe trage, damit der Dreck nicht so an die Hände kommt.“ (B30) | “I have a horse. I ride. I always have to make sure that I wear gloves so that the dirt doesn't get on my hands." (B30) |
| „Im Grunde genommen war das gleich dieser Job-, dieser Wechsel zu einem, zu einem neuen Job bei einem neuen Arbeitgeber mit mit völlig neuen Teilen, neuen Materialien, die ich auf einmal in der Hand hatte.“ (B21) | "Basically, that was this job-, this change to a, to a new job with a new employer with with completely new components, new materials that I had in my hand all of a sudden." (B21) |
| **Timeline** |  |
| „Dann geht es eine ganze Zeitlang gut. Und dann kommt sozusagen wieder ein Rückfall, dass die Hände dann wieder schlechter werden.“ (B1) | “And then it works out for a while. And then there is a relapse, so to speak, and the hands get worse again.” (B1) |
| **Symptoms** | |
| “Innerhalb von ein, zwei Stunden waren kleine Bläschen da, die stark gejuckt haben. Und ja, und dann ging das halt ziemlich schnell, dass das also nicht mehr auszuhalten war, weshalb ich dann einen Arzt aufgesucht habe.“ (B1) | "Within one or two hours, there were small blisters that itched a lot. And then it just went pretty quickly, it wasn’t bearable, which is why I then went to see a doctor." (B1) |
| “Bis sie rissig geworden sind, Rhagaden, blutig, die Abschuppung.” (B3) | "Until they cracked, rhagades, bloody, desquamation." (B3) |
| “Und die Hände, die sahen tatsächlich aus, also ich konnte mir jeden Morgen hier so ein Stück Haut abziehen. Die hing in Fetzen runter.” (B4) | "My hands, they actually looked like- I could peel off a piece of skin just like that every morning. It was hanging down in shreds." (B4) |
| „(…) und durch die Risse haben sich offene Wunden entwickelt und im Nachhinein kam dann halt Hornhaut und selbst bei einem Stift festhalten und was schreiben wollen, ist es wieder aufgeplatzt.” (B10) | "(...) and because of the cracks there were open wounds and after that there was only callus and even if you had a pen in your hand and wanted to write something, it burst open again." (B10) |
| „Die Nägel sind halt nicht so, wie man sie gerne hätte. Die sind verformt. Aber das waren sie schon länger.“ (B13) | "The nails are just not the way you want them to be. They're deformed. But they've been that way for a while." (B13) |
| „Mit Pocken, jucken, eitern, klebriger Flüssigkeit oder eitern jetzt nicht aber eher klebriger Flüssigkeit.” (B19) | "With pox, itching, festering, sticky fluid or- well not festering but rather sticky fluid." (B19) |
| “(…) einen Morgen [war das] so schlimm, dass ich aufgestanden bin, und das war feuerrot, offen“ (B2) | "(...) it was really so bad one morning so I got up, and it was fiery red, open" (B2) |
| „Ist halt nervig, der Juckreiz vor allen Dingen.“ (B22) | "It's just annoying, the itching above all things." (B22) |
| “immer mit Schwellungen, mit Rötungen, wirklich dick“ (B2)  „Die Hautoberfläche war alles wieder schön verheilt. Die war wieder intakt. Aber in der Tiefe hat man immer noch diese Infektion-, äh Entzündungsherde gesehen.“ (B3)  „Also gibt es richtige Risswunden, die, bis das Blut kommt, sozusagen.“ (B8) | "always with swelling, redness, really thick" (B2).  "The surface of the skin was all nicely healed again. It was intact again. But in the depth you still saw these infection-, uh inflammations." (B3)  "So there are real lacerations, until the blood comes, so to speak." (B8) |
| **Controllability** | |
| „Die [Anmk. d. Autor*innen: Ärzt*innen] waren schon recht streng. War ich so nicht gewohnt, aber war dann wohl auch der richtige Weg. Das hat gut geholfen. Das hat schnell angeschlagen.” (B13)  „Da achtet man schon viel mehr drauf, habe ich auch die Handschuhe an. Das habe ich ja alles, aber trotzdem wegen diesen ganzen Handschuhen und super toll aufpassen, es passiert jetzt doch immer.“ (B6) | "They [authors note: doctors] were quite strict. I wasn't used to that, but it was probably the right way to go. That helped a lot. It had a quick effect." (B13)  "Then you pay much more attention to it, I also wear the gloves. I have all that, but still because of all these gloves and super great paying attention, it still happens, all the time." (B6) |
| „Und da fand ich eigentlich, dass die Hände ganz gut aussahen, sie waren ein bisschen gerötet, ein bisschen überwärmt, aber ich kenne es eben halt auch schlimmer.“ (B20) | "And I actually thought that the hands looked quite good, they were a little red, a little overheated, but I'm used to something worse." (B20) |
| „Ansonsten viele Cremes, verschiedene Kortisonstärken, je nach Zustand, wie extrem es gerade ist. (B20) | "(...) lots of creams, different strengths of cortisone, depending on the condition, how extreme it is at that point. (B20) |
| „Aber ich habe […], mit meiner Frau so drüber gesprochen, dass ich einfach diese Feuchtarbeiten einfach alle weglasse.“ (B24) | “I talked to my wife about it like that, that I just leave out all these wet work at home." (B24) |
| „Ich habe geguckt im Geschäft, dass ich oft Handschuhe trage, dass ich die auch nicht zu lange trage. Dass ich nicht drin schwitze und so weiter.“ (B32) | "I wear gloves, but I don't wear them too long either, so that I don't sweat in them and so on." (B32) |
| “Bin beim Hautarzt sehr oft. Also, alle drei, vier Wochen, habe ich Termin zum nachgucken.“ (B9) | "I’m at the dermatologist quite often. So, every three, four weeks, I have appointments to check my skin." (B9) |
| **Consequences** | |
| "Ja, man, es ist halt, man lernt damit zu leben, irgendwann. Ja, man lernt damit zu leben." (B25) | "Yeah, you, it's just, you learn to live with it, eventually. Yeah, you learn to live with it." (B25) |
| „Und wenn ich zum Beispiel einkaufen gehe, dann habe ich jetzt auch inzwischen die Baumwollhandschuhe an. Musste mir allerdings auch schon, auch im Bekanntenkreis, anhören: ‚Oh, Michael Jackson‘ Also dumme Sprüche. Und ja. Und dann kommt natürlich auch ein bisschen Schamgefühl.“ (B7) | "And when I go shopping, for example, I also started wearing cotton gloves. But I've also had to listen to people, also acquaintances, saying: ‘Oh, Michael Jackson.’ Stupid comments. And yeah, and then, of course, there's also a bit of shame." (B7) |
| "Wenn ich aber sage, „Ich nehme die Händedesinfektion.“ hören Sie mich bis am Ende der Straße schreien.“ (B19) | "But if I say, ‘I'll use hand sanitizer.’ you'll hear me screaming in pain all the way down the street." (B19) |
| „Wenn die Hände wirklich offen sind, dann kommt halt auch dazu, dass man irgendwann das Gefühl bekommt, okay, man ist unzuverlässig, weil man einfach sich krankmelden muss, dann wenn es wirklich schlimm ist. (…) Dann habe ich halt immer Angst, dass man denkt, ich bin vielleicht nicht zuverlässig oder dass mir das halt ein Strick draus ziehen könnte.“ (B2) | "When the hands are really open, you also get the feeling at some point that you are unreliable, because you just have to call in sick when it's really bad. (...) Then I'm just always afraid that people will think that maybe I'm not reliable or that it could be used against me." (B2) |
| „Dann fingen die anderen auch an, die Arbeitskollegen. Und die sitzen in einem Frühstücksraum und denn war auch nicht schön. Da sagten sie: ‚Setz dich woanders hin. Das sieht nicht schön aus.‘ Ich sage: ‚Ich kann aber auch nichts daran machen. Es ist eben so.‘ Dann haben die gesagt: ‚Dann zieh irgendwas über.‘ Und dann bin ich da angefangen und habe da im Frühstücksraum schon dann andere Handschuhe angezogen.“ (B34) | "Then the others started too, the work colleagues. And they were sitting in a breakfast room and it wasn't nice. They said, ‘Sit somewhere else. This doesn't look nice.’ I say, ‘But I can't do anything about it. It's just the way it is.’ Then they said, ‘Then put something on.’ And then I started wearing gloves in the breakfast room." (B34) |
| „Ich bin jetzt gerade Oma geworden. Es ist halt schon ein bisschen komisch, wenn man so das Kind, fasse ich das jetzt an, fasse ich das jetzt nicht an? Und halt partnermäßig, ja da habe ich manchmal auch so das Gefühl, streichele ich jetzt meinen Mann oder sagt er: "Nimm deine rauen Hände weg." (B5) | "I’ve just become a grandmother. It's just a bit strange when you're like, ‘Do I touch this child now or not?’ And when it comes to my partner, I sometimes have the feeling- Do I stroke my husband or will he say, ‘Take your rough hands off me?’” (B5) |
| **Coherence** | |
| “Hoffentlich geht das weg oder hoffentlich ist das nicht unheilbar oder so.“ (B12) | "Hopefully it will go away or hopefully it's not incurable or something like that." (B12) |
| „Eigentlich, ja. Weil ich ja auch nicht wei-, ich weiß ja nicht woran es liegt. Dann kann ich auch nicht verstehen so, wa- woher oder warum oder weshalb das kommt. Oder was es ausgelöst hat, dass die Hände so schuppig sind.“ (B28) | "Actually, yes. Because I also don't know-, I don't know what it is. Then I can also not understand so, wha- or why or why. Or whatever triggered it that the hands are so scaly." (B28) |
| “Ach, das ist schon nachvollziehbar. Insofern, dass ja überall in vielen Produkten sich Substanzen verbergen, die gerade in der heutigen Zeit, wo sowieso viele Leute unter allergischen Reaktionen leiden, so was auftreten kann. Also es ist jetzt nicht so, dass ich jetzt völlig verdattert bin, so: ‚Will mich der liebe Gott strafen oder so?‘ (Lacht.) Oder so wie-. Ich habe schon ein gutes Vorstellungsvermögen von so was.” (B12) | "It's quite understandable. In so far as substances are hidden everywhere in many products, especially today, where many people suffer from allergic reactions anyway. So, it's not that I'm completely puzzled like: ‘Does the good Lord want to punish me or something?’ (Laughs.) Or something like-. I already have a good imagination of something like that." (B12) |
| **Emotional representation** |  |
| „Aber bei den Kindern habe ich es halt schon versucht so gut es geht zu verstecken. Zumindest vor den ganzen kleinen. Weil der ist halt auch schon gekommen und hat gesagt ‚Papa, deine Hand sieht ekelig aus.‘“ (B15) | “But I’ve tried to hide it as well as possible from my children. At least from the little ones. Because he has already come and said 'Dad, your hand looks disgusting.'” (B15) |
