# Supplementary material for: Illness perceptions of occupational hand eczema in German patients based on the common-sense model of self-regulation: A qualitative study
Source: PLoS One. 2023 May 12;18(5):e0285791. doi: 10.1371/journal.pone.0285791 (PMC10180686; doi:10.1371/journal.pone.0285791)
Supplement: Attachment — Submitted filename: Comment.docx [file pone.0285791.s005.docx]

Thank you authors for conducting your research on interesting title “Illness perceptions of occupational hand eczema in German 4 patients based on the common-sense model of self-regulation: A qualitative study”. It would benefit for taking action to improve occupational health for work safety. Please find below my comments to improve quality of the paper before publication.

**Abstract:**

- Which qualitative study design was applied in this study?
- It is not clear how you analyzed the data. Please provide more description, including any software used to manage data analysis.
- On the method section you mentioned thirty sex individuals were interviewed, but on the result section, thirty five transcripts were transcribed verbatim and analyzed. Why?
- The first two sentences of the result part need to be written under method part.
- Things written under discussion section are not consistent with what was written under method and result part. Why it is needed further qualitative study once you included all dimensions of the model?

**Introduction:**

- Well-written!

**Methods**

- Study design: Not clear. Which study design you employed?
- Recruitment: Statements written here contained concepts that would be incorporated under ethical considerations. Rather write, in detail, about the sampling technique (s) and criteria’s considered to recruit participates. Merge sampling and recruitment together and describe in detail.
- Data collection: It is surprising that you conducted a 6 minute interview. How did you see it? What is the minimum interview time? You mentioned that the guide was prepared in relation to the dimensions of the model. So, how you see it. This needs especial attention. Additionally, how you fixed the sample size? Were participants come from one organization of different? This is because; different organizations might have different occupational health hazards, which in turn, vary your sample size to reach saturation.
- Trustworthiness: Your paper lacks trustworthiness section, which is one of the most important sections in qualitative research report.

**Result:**

- Please provide detail socio-demographic characteristics of the participants.
- Please provide quote (s) to each categories or themes.

**Discussion:**

- Please provide implication or interpretations for the findings.

**Conclusions**

- Please rewrite the conclusion section to be consistent with your research findings. Write what you understood from the findings.
